# Genome-wide analysis of the JAZ subfamily of transcription factors and functional verification of *BnC08.JAZ1-1* in *Brassica napus*

**DOI:** 10.1186/s13068-022-02192-0

**Published:** 2022-09-12

**Authors:** Ying Wang, Na Li, Jiepeng Zhan, Xinfa Wang, Xue-Rong Zhou, Jiaqin Shi, Hanzhong Wang

**Affiliations:** 1 Oil Crops Research Institute of the Chinese Academy of Agricultural Sciences, Key Laboratory of Biology and Genetic Improvement of Oil Crops, Ministry of Agriculture and Rural Affairs, Wuhan, China; 2 The Laboratory of Melon Crops, Zhengzhou Fruit Research Institute of the Chinese Academy of Agricultural Sciences, Zhengzhou, Henan Province China; 3 Hubei Hongshan Laboratory, Wuhan, China; 4 Commonwealth Scientific & Industrial Research Organisation (CSIRO) Agriculture &Food, Canberra, ACT Australia

**Keywords:** *Brassica*, *JAZ*, Stress, Phytohormone, Seed weight

## Abstract

**Background:**

JAZ subfamily plays crucial roles in growth and development, stress, and hormone responses in various plant species. Despite its importance, the structural and functional analyses of the JAZ subfamily in *Brassica napus* are still limited.

**Results:**

Comparing to the existence of 12 *JAZ* genes (*AtJAZ1*-*AtJAZ12*) in *Arabidopsis*, there are 28, 31, and 56 *JAZ* orthologues in the reference genome of *B. rapa*, *B. oleracea,* and *B. napus*, respectively, in accordance with the proven triplication events during the evolution of *Brassicaceae*. The phylogenetic analysis showed that 127 JAZ proteins from *A. thaliana*, *B. rapa*, *B. oleracea,* and *B. napus* could fall into five groups. The structure analysis of all 127 JAZs showed that these proteins have the common motifs of TIFY and Jas, indicating their conservation in *Brassicaceae* species. In addition, the cis-element analysis showed that the main motif types are related to phytohormones, biotic and abiotic stresses. The qRT-PCR of the representative 11 *JAZ* genes in *B. napus* demonstrated that different groups of *BnJAZ* individuals have distinct patterns of expression under normal conditions or treatments with distinctive abiotic stresses and phytohormones. Especially, the expression of *BnJAZ52* (*BnC08.JAZ1-1*) was significantly repressed by abscisic acid (ABA), gibberellin (GA), indoleacetic acid (IAA), polyethylene glycol (PEG), and NaCl treatments, while induced by methyl jasmonate (MeJA), cold and waterlogging. Expression pattern analysis showed that *BnC08.JAZ1-1* was mainly expressed in the vascular bundle and young flower including petal, pistil, stamen, and developing ovule, but not in the stem, leaf, and mature silique and seed. Subcellular localization showed that the protein was localized in the nucleus, in line with its orthologues in *Arabidopsis*. Overexpression of *BnC08.JAZ1-1* in *Arabidopsis* resulted in enhanced seed weight, likely through regulating the expression of the downstream response genes involved in the ubiquitin–proteasome pathway and phospholipid metabolism pathway.

**Conclusions:**

The systematic identification, phylogenetic, syntenic, and expression analyses of BnJAZs subfamily improve our understanding of their roles in responses to stress and phytohormone in *B. napus*. In addition, the preliminary functional validation of *BnC08.JAZ1-1* in *Arabidopsis* demonstrated that this subfamily might also play a role in regulating seed weight.

**Supplementary Information:**

The online version contains supplementary material available at 10.1186/s13068-022-02192-0.

## Background

Oilseed rape (*Brassica napus L.*) is one of the important industrial crops and the third largest source of vegetable oil in the world [1]. Oilseeds are not only an agricultural product necessary for human daily life, but also an important industrial raw material for bioethanol and biodiesel [2, 3]. With the rapid increase in the global population, the situation of energy supply and demand is becoming tenser. The diversification, reproducibility, and cleanliness of energy have been the inevitable choice for the development of human society. As new alternative energy, rapeseed biodiesel is rapidly developing in countries all over the world [4, 5]. Improving oilseed yield can relieve the pressure on bioenergy demand. Although three components of oilseed yield per plant (silique number per plant, seed number per silique, and seed weight) show different degrees of negative correlation, their correlation coefficients are generally small [6, 7], indicating that yield can be improved by increasing the individual yield components (such as seed weight).

Jasmonic acid (JA) and its derivative jasmonates (JAs) play a vital role in response to adversity stress such as pathogen invasion and wounds [8, 9, 10, 11, 12, 13]. In addition, JAs also contribute to development and growth including the regulation of pollen development [14, 15, 16], stamen development [15, 16], flowering time [17, 18], root growth [19], leaf senescence [20, 21], and so on. Therefore, JA and JAs are considered to be regulators that maintain the balance between plant growth and defense.

It is well known that JA signaling transduction requires the involvement of JAZ (JASMONATE-ZIM DOMAIN) proteins, which act as a transcriptional repressor protein [22, 23, 24, 25, 26]. JAZ subfamily is a member of the TIFY transcription factor superfamily, which possesses two conserved functional domains, TIFY (also known as ZIM) and Jas (CCT_2). In the JA signal pathway, the TIFY domain recruit NINJA (Novel Interactor of JAZ) and TPL (TOPLESS) co-repressors and interact with downstream transcription factors to repress the expression of JA response genes [23, 27, 28, 29]. The Jas domain can interact with MYC2 or COI1, respectively, which then inhibits or activates the expression of downstream responsive genes, relying on the low or high level of JA [23, 24, 30].

Previous studies indicate that the *JAZ* genes regulate plant growth and development and its response to adversity stresses [31, 32, 33, 34, 35, 36]. In addition, the different JAZ members may have different biological functions. For example, the overexpression of *JAZ1* or *JAZ4* in *Arabidopsis* reduces its tolerance to freezing stress [36]. OsJAZ1 can interact with OsMYC2, which activates OsMADS1’s role in regulating spikelet development in rice [37]. The single mutants *jaz4-1*, *jaz7*-*1*, and *jaz8-1* expedite dark-induced leaf senescence, whereas *jaz6-1* and *jaz6-2* retard leaf senescence [20, 21, 38]. The *jaz6-18* and *jaz8-10* double mutant have shown retarded root growth and enhanced resistance to the necrotrophic fungus *Botrytis cinerea* [39]. Except for their major role in JA signaling, JAZ protein is also involved in other hormone signaling pathways by binding to a variety of transcription factors, thereby co-regulating plant growth, development, and hormone response [9, 40]. For example, the direct interplay between JAZs and DELLAs mediates the antagonistic interaction between JA and GA [41, 42]. The expression of genes involved in cytokinin responses is largely activated by JA or JA-dependent stress responses [43]. JA controls the differentiation of xylem in *Arabidopsis* roots, and JA-dependent xylem development is closely related to the antagonistic interplay between JA and cytokinin [44]. The interplay between JA and auxin is also involved in multiple aspects of plant development including root development, flower development, and leaf senescence [21, 45, 46, 47, 48].

The previous studies of the JAZ subfamily have focused on gene identification, expression response to various hormones or stress treatments in multiple model plants (e.g., *Arabidopsis*), and crops (e.g., rice, maize, wheat, and soybean) [49, 50, 51, 52, 53]. Although several studies on the TIFY family have been reported in *B. rapa*, *B. oleracea,* and *B. napus* separately, there is still a lack of systematic characterization and comparatively evolutionary study across these three species and *A. thaliana* with a focus on the JAZ subfamily. In this study, we identified the JAZ subfamily with 56 genes in *B. napus*, and investigated their physicochemical characteristic, gene structure, cis-element, motif composition, phylogenetic and syntenic relationship. We also analyzed the tissue-specific expression patterns of BnJAZ genes with a focus on their response to several phytohormones and stresses. In addition, a representative gene *BnJAZ52* (named *BnC08.JAZ1-1*) that was differentially expressed between two pools of large and small seeds, was over-expressed in *Arabidopsis* to preliminarily verify its function. These results provided a systematic view on the evolution and function of *JAZ* genes in *Brassica*.

## Results

### Identification and phylogenesis of the JAZ subfamily in *Brassica napus*

A total of 56, 28, and 31 orthologues were identified from the reference genomes of *B. napus* as well as its two progenitors *B. rapa* and *B. oleracea*, respectively (Table 1). The characteristic of these genes, including the length of coding sequence, the molecular weight of protein, isoelectric point, and subcellular localization were analyzed. The length of 56 identified *BnJAZ* genes in *B. napus* showed a wide range from 556 to 9131 bp, indicating their large variation. The proteins of 56 BnJAZs ranged from 116 (BnJAZ40) to 564 (BnJAZ53) amino acid residues, with molecular weights of 13.13 kDa to 62.22 kDa. The predicted isoelectric points ranged from 5 (BnJAZ4) to 10 (BnJAZ56). Except for five BnJAZ proteins localized in chloroplast, four in the cytoplasm, one each in the endoplasmic reticulum, Golgi apparatus, or mitochondria, other 44 proteins were predicted to be located in the nucleus, indicating that most of them might be transcription factors.

**Table 1:** Characterization of the 56 BnJAZ genes identified in *Brassica napus*

Gene name	Gene ID	Genomic position (bp)	CDS length (bp)	Exon number	Molecular weight (KDA)	Isoelectric point	Subcellular prediction
BnJAZ1	BnaA01G0203400ZS	A01:12583796–12585951: +	963	8	35.09526	9.12	Nucleus
BnJAZ2	BnaA01G0308500ZS	A01:28824803–28828698: +	1104	9	40.68164	6.84	Chloroplast
BnJAZ3	BnaA01G0331000ZS	A01:30506077–30510006:-	1062	7	37.57904	9.3	Nucleus
BnJAZ4	BnaA02G0000800ZS	A02:293262–294602: +	651	5	22.47421	4.96	Cytoplasm
BnJAZ5	BnaA02G0001900ZS	A02:350665–352002:-	534	6	19.35639	9.82	Cytoplasm
BnJAZ6	BnaA02G0047200ZS	A02:2647926–2649211:-	639	3	23.99492	9.95	Golgi apparatus
BnJAZ7	BnaA02G0190100ZS	A02:11734586–11736653:-	825	7	29.34219	9.86	Nucleus
BnJAZ8	BnaA02G0200100ZS	A02:12545103–12546473:-	822	4	30.1618	9.11	Nucleus
BnJAZ9	BnaA02G0213500ZS	A02:13411661–13412862: +	723	5	26.19755	9.21	Nucleus
BnJAZ10	BnaA03G0052500ZS	A03:2524449–2525938: +	672	4	25.24727	9.81	Chloroplast
BnJAZ11	BnaA05G0380600ZS	A05:37865369–37867939: +	1011	7	35.87111	9.51	Nucleus
BnJAZ12	BnaA06G0119400ZS	A06:6986490–6987776:-	801	4	29.8044	9.18	Nucleus
BnJAZ13	BnaA06G0133300ZS	A06:7845736–7847029:-	765	4	27.30006	9.85	Chloroplast
BnJAZ14	BnaA07G0249100ZS	A07:24141740–24144675:-	675	5	24.5857	9.14	Nucleus
BnJAZ15	BnaA07G0266400ZS	A07:25167684–25168787:-	639	5	23.40839	9.03	Mitochondria
BnJAZ16	BnaA07G0320600ZS	A07:28617818–28620360: +	801	7	28.64052	9.8	Nucleus
BnJAZ17	BnaA07G0333200ZS	A07:29319157–29320650: +	825	4	30.59633	9.08	Nucleus
BnJAZ18	BnaA07G0349300ZS	A07:30135515–30136690:-	738	5	26.75717	9.2	Nucleus
BnJAZ19	BnaA08G0021500ZS	A08:1797523–1798971:-	936	6	33.95753	6	Nucleus
BnJAZ20	BnaA08G0090300ZS	A08:15229435–15231385:-	957	8	35.01222	9.13	Nucleus
BnJAZ21	BnaA08G0205900ZS	A08:22909636–22910221: +	393	3	14.97178	9.85	Nucleus
BnJAZ22	BnaA08G0252900ZS	A08:25292226–25293349: +	780	4	28.31018	9.49	Nucleus
BnJAZ23	BnaA08G0262000ZS	A08:25690409–25691687: +	804	4	29.70646	9.11	Nucleus
BnJAZ24	BnaA09G0610300ZS	A09:60116929–60119612: +	822	7	29.82271	9.49	Nucleus
BnJAZ25	BnaA09G0618000ZS	A09:60520671–60529802:-	948	4	35.08154	9.67	Chloroplast
BnJAZ26	BnaA10G0170700ZS	A10:20196301–20197382: +	612	5	21.43097	6.92	Nucleus
BnJAZ27	BnaA10G0223600ZS	A10:22916557–22919394: +	591	5	21.8192	9.91	Nucleus
BnJAZ28	BnaC01G0254700ZS	C01:20032455–20034770:-	945	9	34.53359	8.31	Nucleus
BnJAZ29	BnaC01G0382400ZS	C01:44212689–44217541: +	1161	9	43.03258	8.52	Nucleus
BnJAZ30	BnaC01G0408900ZS	C01:47295575–47298846: +	1062	7	37.52209	9.25	Nucleus
BnJAZ31	BnaC02G0054000ZS	C02:3401968–3404083:-	591	5	21.80012	9.96	Nucleus
BnJAZ32	BnaC02G0104800ZS	C02:6810525–6811864: +	666	5	23.08295	4.97	Cytoplasm
BnJAZ33	BnaC02G0251900ZS	C02:23433804–23435871: +	825	7	29.40624	9.93	Nucleus
BnJAZ34	BnaC02G0266500ZS	C02:25372570–25373915:-	819	4	29.94936	7.07	Nucleus
BnJAZ35	BnaC02G0285000ZS	C02:27147851–27149030: +	723	5	26.20356	9.3	Nucleus
BnJAZ36	BnaC03G0060300ZS	C03:3153036–3154515:-	672	4	25.19816	9.81	Chloroplast
BnJAZ37	BnaC03G0662600ZS	C03:64076368–64076942: +	393	3	14.97178	9.85	Nucleus
BnJAZ38	BnaC03G0663300ZS	C03:64131128–64131740:-	435	3	16.74187	8.75	Nucleus
BnJAZ39	BnaC03G0787400ZS	C03:76176548–76178066: +	891	6	32.35073	5.79	Nucleus
BnJAZ40	BnaC04G0121900ZS	C04:11013009–11013565:-	351	2	13.12705	9.15	Nucleus
BnJAZ41	BnaC05G0147200ZS	C05:9303032–9304540: +	771	5	28.39794	9.69	Nucleus
BnJAZ42	BnaC05G0160800ZS	C05:10437306–10438599: +	765	4	27.25592	9.71	Nucleus
BnJAZ43	BnaC05G0259000ZS	C05:21020991–21021569: +	399	3	15.24115	9.47	Nucleus
BnJAZ44	BnaC05G0424600ZS	C05:47654460–47657298:-	1008	7	35.76295	9.47	Nucleus
BnJAZ45	BnaC06G0274800ZS	C06:38103129–38104304:-	675	5	24.46158	9.23	Nucleus
BnJAZ46	BnaC06G0299300ZS	C06:40408436–40409553:-	639	5	23.31825	9.04	Nucleus
BnJAZ47	BnaC06G0373700ZS	C06:47272615–47275070: +	801	7	28.53251	9.57	Nucleus
BnJAZ48	BnaC06G0391300ZS	C06:48490374–48491996: +	816	4	30.32298	9.47	Nucleus
BnJAZ49	BnaC06G0411600ZS	C06:49662272–49663471:-	741	5	27.23772	8.96	Nucleus
BnJAZ50	BnaC08G0129100ZS	C08:23055859–23058016: +	1089	8	39.79375	8.28	Endoplasmic reticulum
BnJAZ51	BnaC08G0240100ZS	C08:33426315–33427601: +	807	4	29.78851	8.84	Nucleus
BnJAZ52	BnaC08G0251600ZS	C08:34242963–34244270:-	795	4	28.74264	9.65	Nucleus
BnJAZ53	BnaC08G0464200ZS	C08:49511838–49516399: +	1695	6	62.21759	9.18	Nucleus
BnJAZ54	BnaC08G0473200ZS	C08:50094384–50095986: +	1032	4	38.00173	9.49	Nucleus
BnJAZ55	BnaC09G0456500ZS	C09:56962395–56963463: +	606	5	21.25174	6.45	Cytoplasm
BnJAZ56	BnaC09G0528700ZS	C09:62610296–62611889: +	600	5	22.46196	9.99	Nucleus

**Fig. 1:**
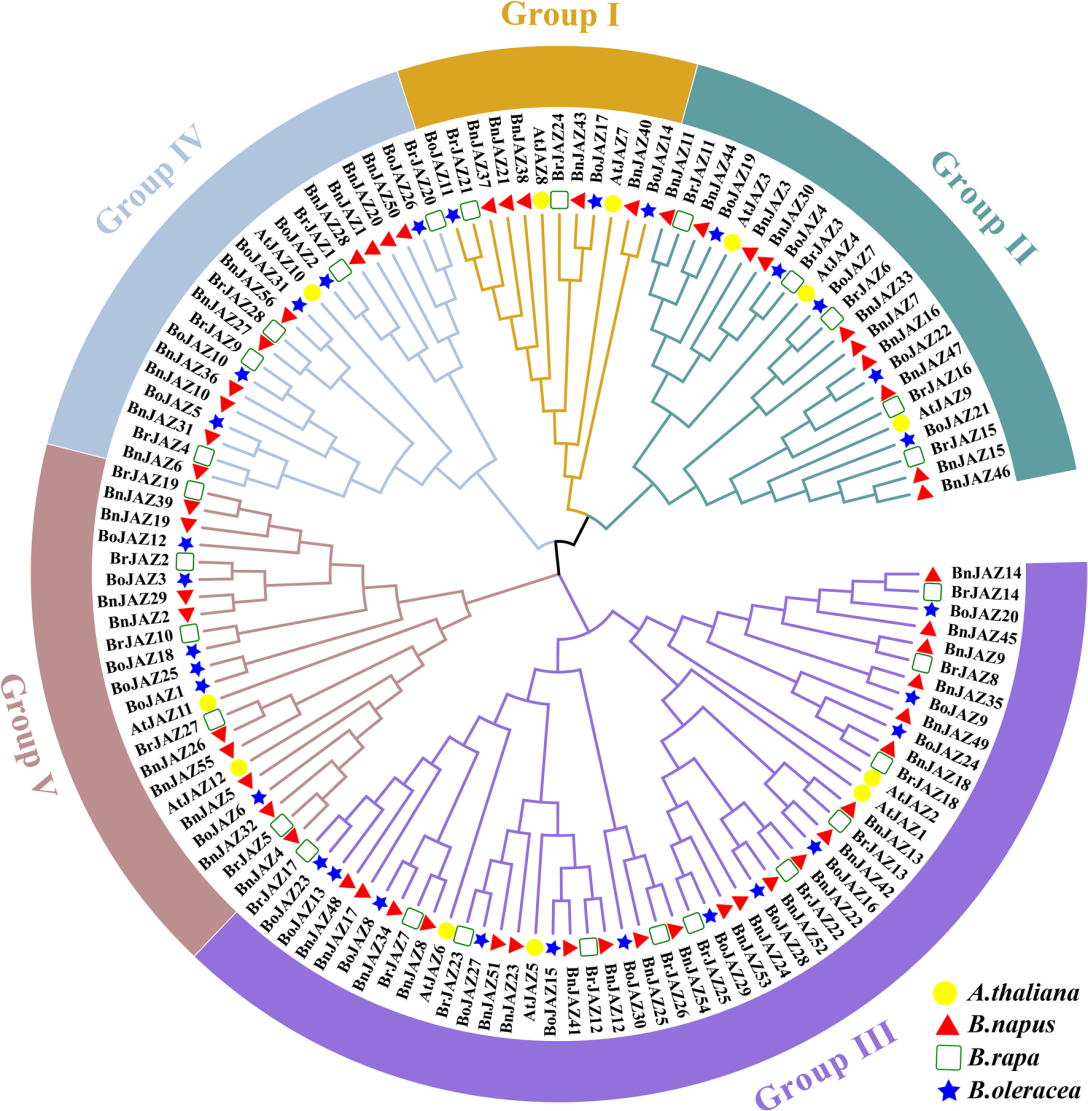
Phylogenetic tree of JAZ proteins from four species in *Brassicaceae*. Overall, 56 BnJAZs (red triangle), 31 BoJAZs (blue star), 28 BrJAZs (green box), and 12 AtJAZs (yellow circle) were classified into five groups (Group I–V) based on domain and 1000 bootstrap values. At: *A. thaliana*; Bn: *B. napus*; Bra: *B. rapa*; Bol: *B. oleracea*

To elucidate the phylogenetic relationships among the *JAZ* gene family, all 127 JAZ proteins (including 56 from *B. napus*, 28 from *B. rapa*, 31 from *B. oleracea,* and 12 from *A. thaliana*) were used to construct a phylogenetic tree (Fig. 1 and Additional file 1: Table S1). All JAZs were distinctly divided into five groups (I, II, III, IV, and V) as reported in *Arabidopsis* [54], which contained 12, 23, 49, 21, and 22 members, respectively. It should be noted that nearly half were classified into Group III, which contained 22 BnJAZs (half were from the A and C sub-genomes), 11 BraJAZs, 12 BolJAZs, and 4 AtJAZs. Except for Group I, the phylogenic relationships among JAZ orthologues were consistent with the evolutionary relationship of their species' origin.

### Gene structure and motif composition of BnJAZ subfamily

**Fig. 2:**
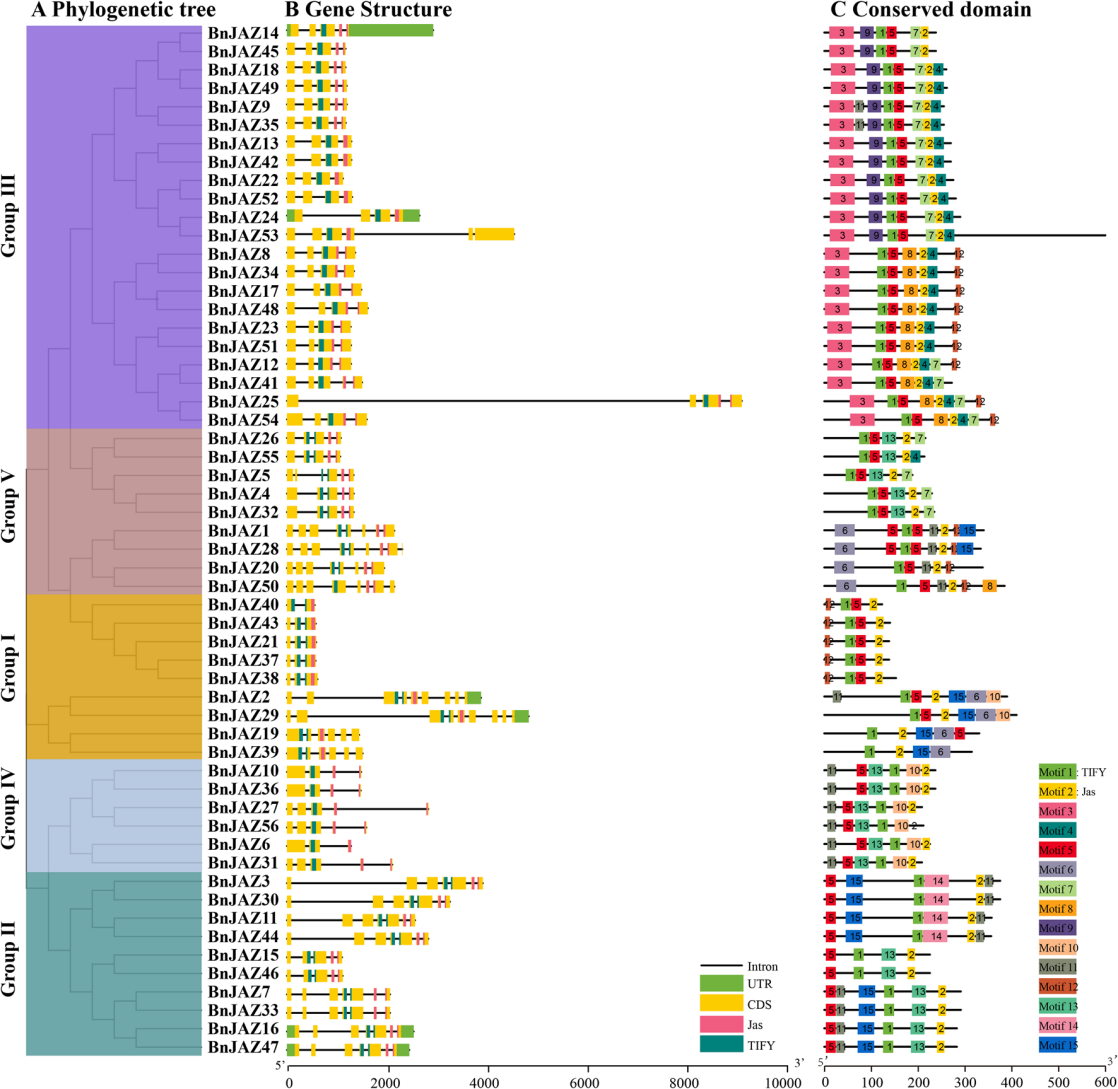
Phylogeny, cis-element, and motif analysis of BnJAZs. **A** The phylogenetic tree of 56 BnJAZs. They are classified into five groups that are distinguished by the different colors. **B** The gene structure of 56 *BnJAZ*s. Different cis-elements are shown with columns in different colors. **C** The conserved motifs were identified in the coding region of 56 BnJAZs. The different-colored boxes represent different motifs

To explore the possible structural evolution of JAZs in *B. napus*, all 56 BnJAZs were analyzed for their gene structure, protein motif composition, and cis-element (Fig. 2 and Additional file 2: Table S2A).

A total of 69 to 193 cis-elements were recognized in the 2-kb upstream regulatory sequence of the 56 *BnJAZ*s genes, which were divided into types 1 to 33 (Additional file 2: Table S2B). Summary statistics of cis-elements number for the different types showed that “core promoter element-TATA box” had the largest number, followed by “light responsive element”, “common cis-acting element-CAAT box” and “short function”; “abscisic acid responsiveness”, “MeJA (Methyl jasmonate) responsive element” and “the anaerobic induction” accounted for a considerable number. There were only a few elements in the other 26 types. It should be noted that the main cis-elements types were involved in phytohormones, biotic or abiotic stress. For the identified 25 types of light responsiveness elements, G box and Box4 had the largest number, followed by GT1, TCT, AE, GATA, MRE, I-box, ATCT, and TCCC, whereas the other 15 types were few. Additionally, several cis-elements were responsive to phytohormones, including abscisic acid (e.g., ABRE), auxin (e.g., TGA-element), gibberellin (e.g., GARE-motif, P-box, TATC-box), MeJA (e.g., CGTCA-motif, TGACG-motif), and salicylic acid (e.g., TCA-element) (Additional file 3: Table S3A). In addition, some cis-elements were responsive to abiotic stress, including low temperature (LTR), drought (MBS), defense and stress (TC-rich repeats), and the anaerobic (ARE) response. Unexpectedly, there was a large difference in the type and number of cis-elements among the different groups or even within the same group.

*BnJAZ* genes possessed two to nine exons, thus correspondingly containing one to eight introns (Fig. 2B). Only one gene (*BnJAZ40*) had two exons and one intron. As expected, all 56 BnJAZ proteins have conserved domains TIFY and CCT_2 (Jas) where the TIFY domain was located at the N-terminal of CCT_2 (Jas). In addition, these two domains might or might not be split by introns, therefore generating four patterns. Interestingly, the TIFY domain of five BnJAZs in Group I was all split by introns, whereas both structural domains of another five BnJAZs in Group V were split by introns.

All BnJAZ proteins were subjected to the MEME motif analysis, and a total of 15 conserved motifs were identified (Fig. 2C and Additional file 3: Table S3). It should be noted that all 56 BnJAZ proteins processed only two common motifs, which were motif 1 and motif 2 (TIFY and CCT_2 domain), suggesting that they were the core domains for the JAZ subfamily. The annotation of the domain using the SMART website showed that the CCT domain controlled photoperiodic flowering and the TIFY domain involved in the regulation of inflorescence and flower development [55, 56]. As expected, the BnJAZ members belonging to the same groups displayed a similar motif composition. For example, motif 3 (NT domain) was unique to group III, whereas motif 14 (unknown domain) was specific to group II. The clustered BnJAZ pairs, i.e., BnJAZ18/49, BnJAZ9/35, showed the same motif distribution. Overall, the *BnJAZ* genes within the same group generally had similar or the same gene structures and protein motif compositions, strongly supporting the reliability of the group classifications.

### Syntenic analysis of *BnJAZ* genes

To explore the evolution of the JAZ subfamily from the comment ancestor, their syntenic relationship was analyzed between *Arabidopsis* and *Brassica* genus (Additional file 4: Table S4). *AtJAZ4* and *AtJAZ11* had no orthologues in *Brassica* and nine *BnJAZ*s (*BnJAZ1, BnJAZ2, BnJAZ19, BnJAZ20, BnJAZ28, BnJAZ29, BnJAZ3, BnJAZ50, BnJAZ53*) have no orthologues in *Arabidopsis*, indicating the regeneration or loss of these genes during evolution. Interestingly, *AtJAZ2, AtJAZ5, AtJAZ7, AtJAZ9, and AtJAZ10* had three orthologues in both *B. rapa* and *B. oleracea*, and were expected for six orthologues in *B. napus*, in line with the previously proved triplication from the common ancestor [57, 58]. *AtJAZ3* and *AtJAZ6* had two orthologues in both *B. rapa* and *B. oleracea* and were expected for four copies in *B. napus*, supporting the proposed hypothesis that triplication was usually followed by diploidization [58].

To elucidate the evolutionary constrictions on the *BnJAZ* family, the Ka, Ks, and their ratio was calculated for the syntenic gene pairs between *B. napus* and *Arabidopsis*/*B. rapa*/*B. oleracea* (Additional file 6: Table S6). The result showed that most (91.0%) of the syntenic *BnJAZ* gene pairs had a Ka/Ks ratio of < 1, indicating a purifying selection pressure. The remaining (9.0%) had a Ka/Ks ratio of > 1, suggesting accelerated evolution under positive selection (Additional file 5: Table S5). For example, the three syntenic gene pairs (*BraJAZ2/BnJAZ2; BraJAZ19/BnJAZ19; BolJAZ3/BnJAZ29*) showed the smallest Ka/Ks value, all of which lost their syntenic relationship with *AtJAZ11* and *AtJAZ12* in the Group V.

**Fig. 3:**
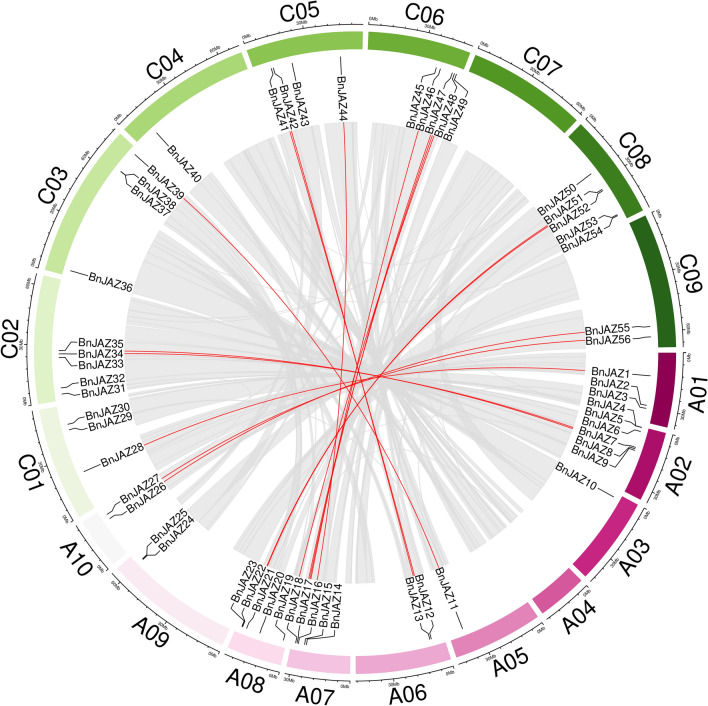
Chromosomal location and inter-chromosomal association of *BnJAZ* genes. The reference genome of Zhongshuang11 is shown in a circle with small gaps to separate the different chromosomes. Gray lines indicate all syntenic blocks in the *B. napus* genome, and the red lines display duplicated *JAZ* gene pairs

Briefly, these 56 *BnJAZ* genes were unevenly distributed on the 17 chromosomes (except for A04 and C07), among them, 27 and 29 were on A and C sub-genomes, respectively. Chromosome A02 contained the largest number of *BnJAZ*s (6), followed by A07, A08, C02, C06, and C08 (5), whereas some chromosomes (e.g., A03, A05, C04) had only one gene. The length of chromosomes was not correlated with the number of *JAZ* genes. Besides, 15 pairs of paralogous genes were recognized between A and C sub-genomes, except for A03, A04, A09, C04, and C07 chromosomes (Fig. 3). It should be noted that all 15 pairs of paralogous genes were located in the syntenic regions, which should originate from the same genomic segments of the common ancestor. Highly accordant with this, no tandem repeat paralogues were found within these genes, therefore they might have originated from whole-genome duplication (WGD) rather than gene proliferation. The chromosomal locations of almost all *JAZ* genes in *B. napus* were similar to their orthologues in *B. rapa* and *B. oleracea*, with a few exceptions. For example, the *BolC07g051380.2J* in *B. oleracea* had no orthologues in *B. napus*. Interestingly, there was no *JAZ* gene distributing on chromosome A04 in *B. rapa* and *B. napus*.

### Expression profiling of the *BnJAZ* genes in main organs/tissues

**Fig. 4:**
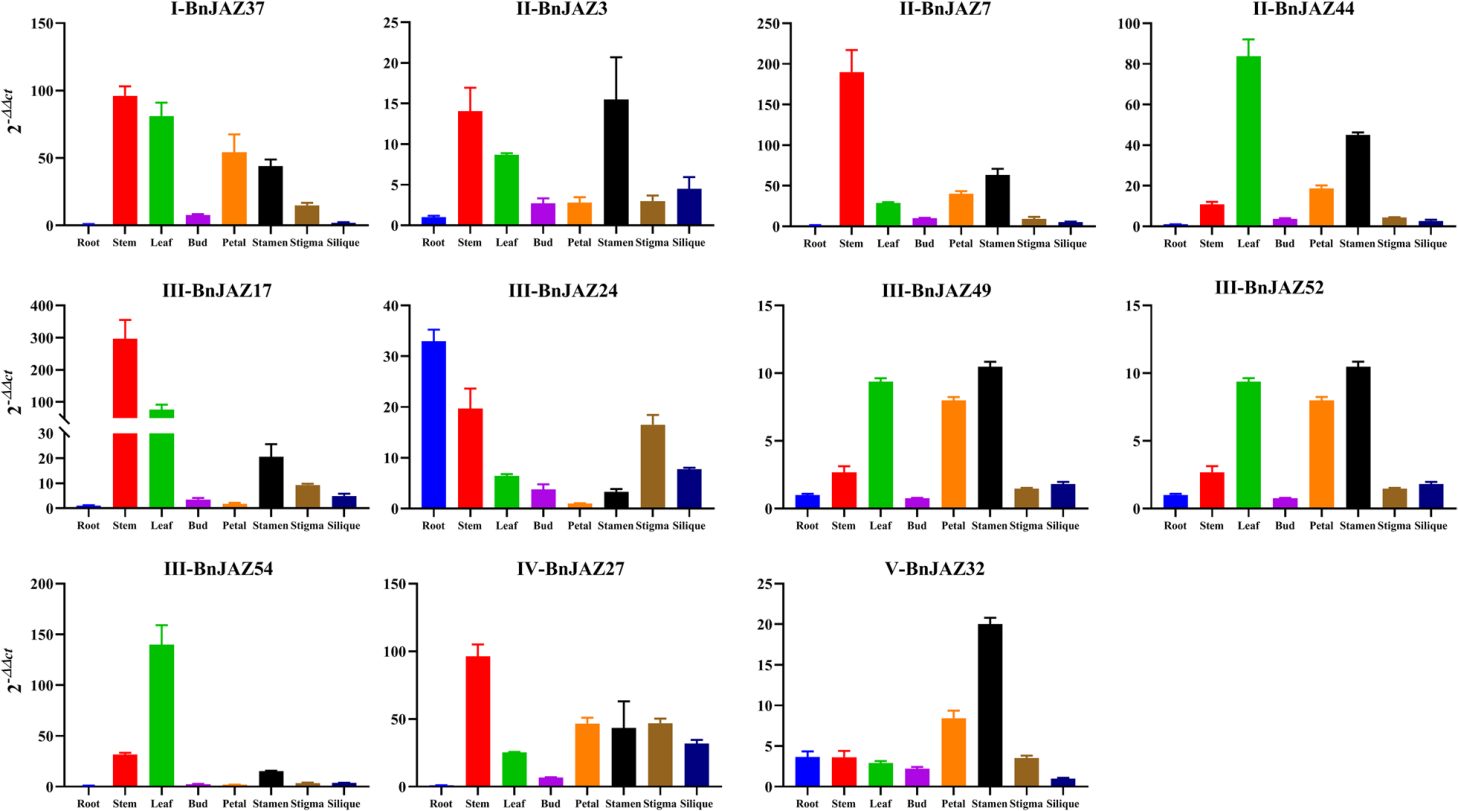
Expression analysis of 11 representative *BnJAZ* genes in eight organs. The horizontal and vertical axes show the different organs and relative expression levels, respectively. Data were normalized to the β-actin gene and vertical bars indicate standard deviation

To predict possible functions through overlapping expression patterns for the *BnJAZ* genes, the expression levels of 11 representative *BnJAZ* genes from five groups were detected in eight different tissues and organs (including root, stem, leaf, bud, petal, stamen, stigma, and silique) by real-time quantitative RT-PCR (Fig. 4). The correlation between the expression levels of 11 *BnJAZ* genes in eight organs and tissues was analyzed. The results showed that high correlations were observed among bud, petal, and stamen, or between silique and stigma. In addition, a similar expression pattern was observed between several pairs of genes, such as *BnJAZ7/17*, *BnJAZ44/49, BnJAZ3/52, BnJAZ44/54, BnJAZ7/27*. Generally, the expression levels of these genes showed great variations in different tissues, which were high in stem, followed by leaf, stamen, and petal, and relatively low in other tissues. In addition, *BnJAZ17, BnJAZ7, BnJAZ37*, and *BnJAZ27* showed relatively high expression, whereas the expression levels of the other five genes were low. Interestingly, all of these genes showed a very low expression in roots, except for *BnJAZ24*, indicating its functional differentiation. Overall, the diverse expression patterns of *JAZ* genes in distinct tissues and organs suggested that these members might play diverse functions.

### Expression pattern of *BnJAZ* genes in response to different abiotic stresses and hormonal treatments

**Fig. 5:**
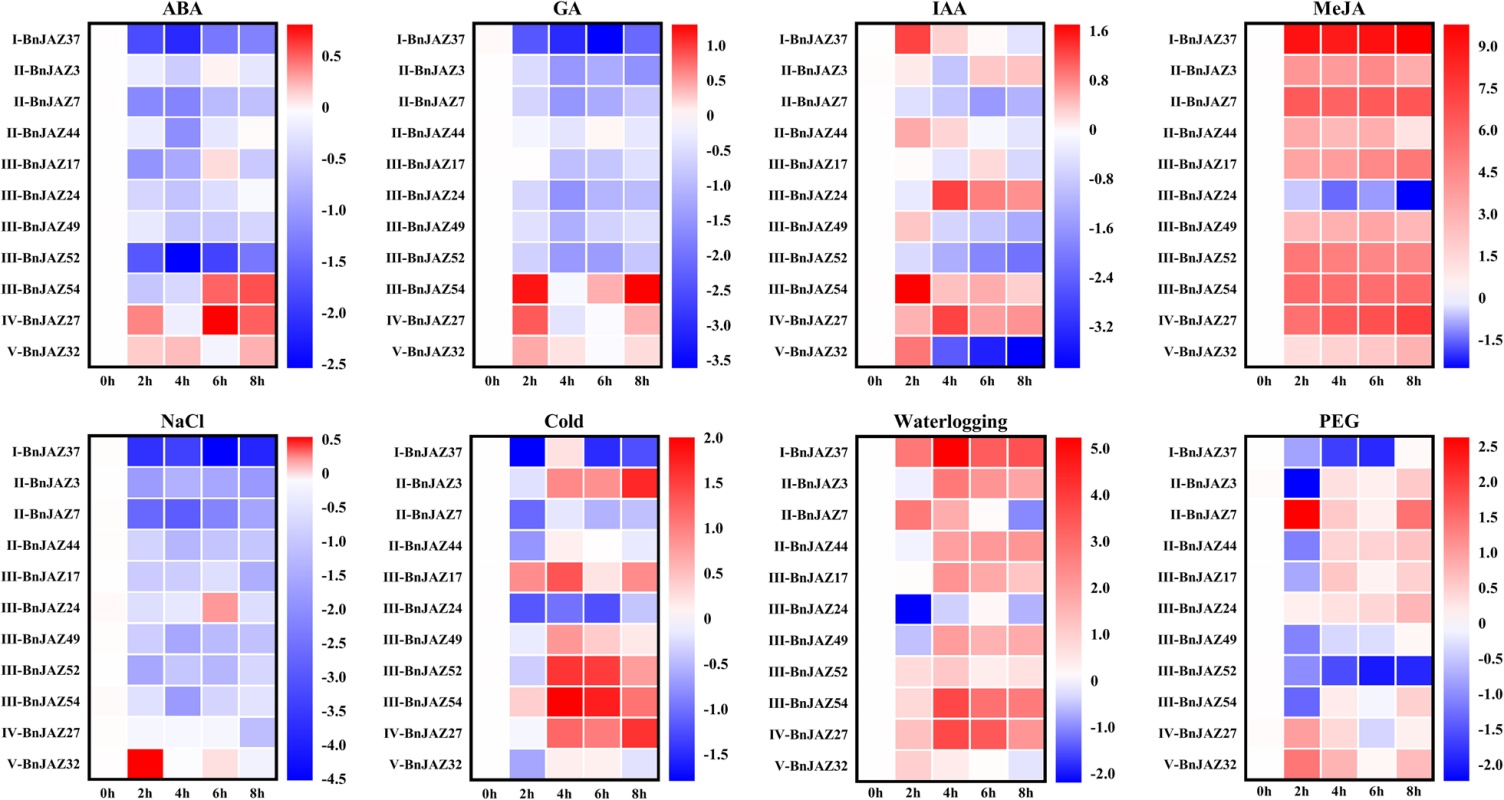
Expression patterns of 11 representative *BnJAZ* genes under various hormones and abiotic stress. The 0 h (CK), 2 h, 4 h, 6 h, and 8 h indicate the time points (hours) when the samples were obtained for expression analysis after different treatments. The colored boxes show the normalized expression level examined via the 2^− ΔΔCT^ method. The legend shows the color columns in which red and blue represent the two extremes of high and low expression, respectively

Based on the cis-elements analysis, these *BnJAZ* genes were predicted to be involved in the response to abiotic stresses and phytohormones. To further confirm this hypothesis, the expression levels of the above-mentioned 11 representative *BnJAZ* genes from five groups were detected after different treatments (PEG, NaCl, cold, waterlogging, ABA, GA, MeJA, and IAA) (Fig. 5). Generally, all 11 *BnJAZ* genes were significantly induced or repressed by multiple treatments, in line with their functional prediction by cis-element analysis. Especially, among eight treatments, MeJA showed the largest effect on the expression of these *BnJAZ* genes, in line with their most important core function domain of Jas. Interestingly, MeJA and waterlogging treatment tended to induce the expression of these *BnJAZ* genes except for *BnJAZ24*, while NaCl likely repressed their expression. ABA and GA showed similar effects on the expression of these genes, which repressed the expression of eight *BnJAZ* genes in Groups I to V except for *BnJAZ27*, *BnJAZ32,* and *BnJAZ54*. Under the treatment of IAA, cold, and PEG, the expressions of 11 *BnJAZ* genes could be divided into four patterns, i.e., a gradual increase or decline, an initial increase followed by a reduction, or an initial reduction followed by an increase.

Obviously, none of the 11 genes showed consistent performance in expression patterns under all eight treatments. Especially, several genes showed opposite expression patterns between the different treatments. For example, the expression of *BnJAZ24* was remarkably up-regulated after 4 h of the IAA treatment, while down-regulated under the treatments of the other three phytohormones ABA, GA, and MeJA. For instance, *BnJAZ37* was significantly repressed by ABA and GA, whereas displayed substantially higher expression at 1–8 h under MeJA treatment. Under the abiotic stimuli, *BnJAZ37* was rapidly repressed by NaCl, cold, and PEG stress, but was up-regulated under waterlogging stress. *BnJAZ52* was significantly repressed by ABA, GA, IAA, PEG, and NaCl treatments, while induced under MeJA, cold, and waterlogging.

### Overexpression of *BnJAZ52* (*BnC08.JAZ1-1*) increased seed weight in *Arabidopsis*

Under the hormone treatment, *BnJAZ52* was up-regulated by MeJA, whereas repressed by GA, ABA, and IAA suggesting that *BnJAZ52* particularly took part in the JA signal pathway, same as its orthologue *AtJAZ1* in *Arabidopsis*. In addition, a previous study showed that JA signaling pathway was involved in seed development and size [12, 59–61]. More importantly, the expression of *BnJAZ52* in large-seed lines was about twice as in small-seed lines in a previously reported RNA-seq study using bulked seeds at 25 days after flowering [66]. These results highly suggested that *BnJAZ52* might also have a role in regulating seed size. Therefore, *BnJAZ52* was selected for further experimental exploration. As *BnJAZ52* was homologous to *JAZ1* in *Arabidopsis* and had two orthologues on the C08 chromosome of *B. napus*, it was designated as *BnC08.JAZ1-1* hereafter.

**Fig. 6:**
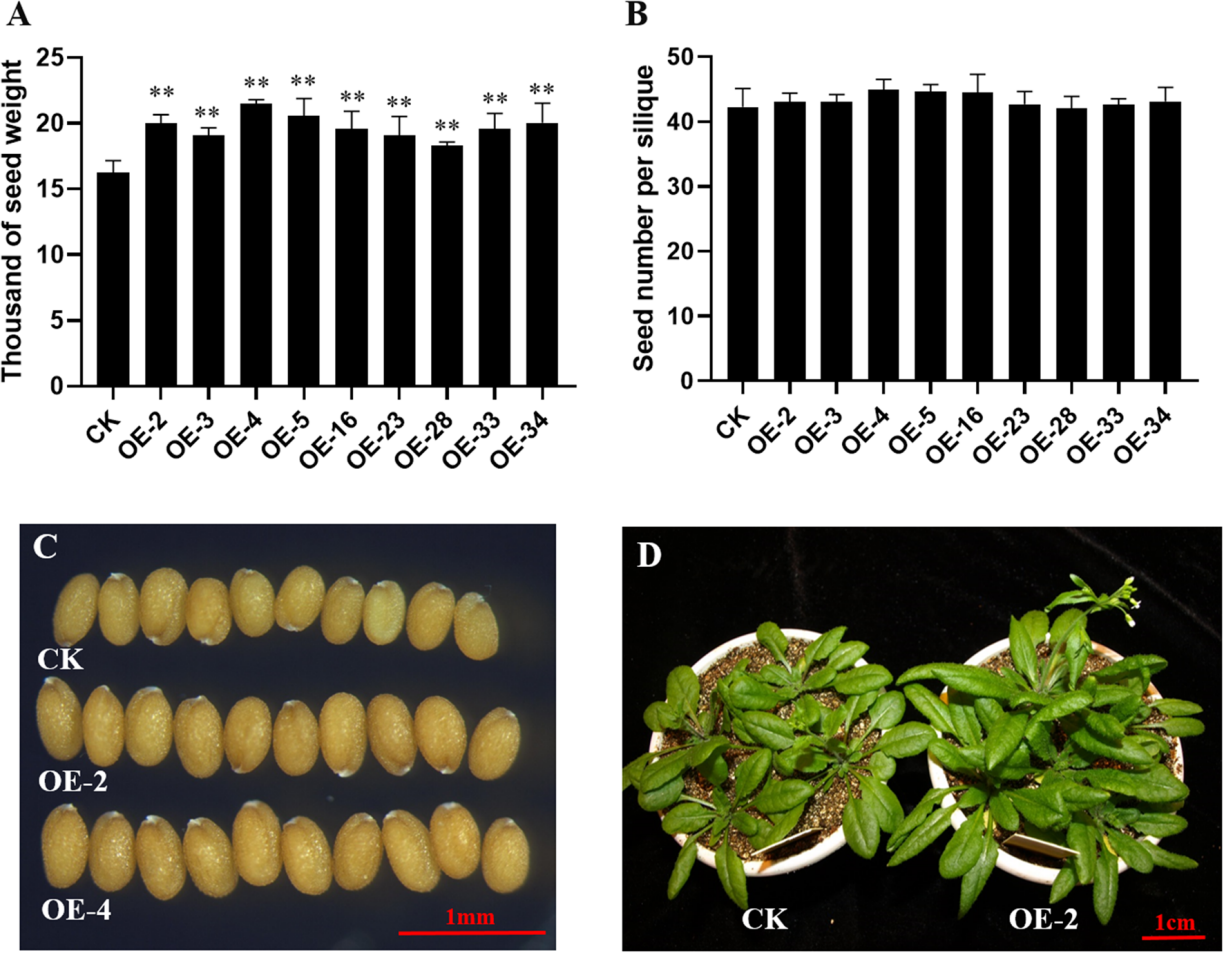
Phenotypes of *BnC08.JAZ1-1* overexpression transgenic *Arabidopsis* plants. **A** Thousand-seed weight of overexpression lines compared to control (CK). **B** Comparison of seed number per silique (SNPS) between overexpression lines and CK. **C** The seed size of overexpression lines compared to CK at the mature stage. **D** The flowering time of overexpression lines compared to CK during the transition stage from vegetative to reproductive growth. Note: *, **, and *** represent the significant level at *P* = 0.05, 0.01, and 0.001, respectively, as determined by Student’s *t*-test

To understand the function of *BnC08.JAZ1-1*, its CDS sequence was cloned from Zhongshuang11 and over-expressed in *A. thaliana*. The phenotypes of transgenic lines were investigated. At the seedling stage, *BnC08.JAZ1-1*-OE plants flowered about 3 days earlier than the control (Fig. 6D). At the mature stage, the seed weight of nine independent transgenic lines in the T3 generation significantly increased, with the proportions from 17.4% to 27.2% compared to control (Fig. 6A, C). However, there was no significant difference in seed number per silique between transgenic lines and control (Fig. 6B). These results suggested that *BnC08.JAZ1-1* was a vital positive regulator that promotes plant growth and development, especially for flowering time and seed weight.

### Expression pattern and subcellular location localization of *BnC08.JAZ1-1*

**Fig. 7:**
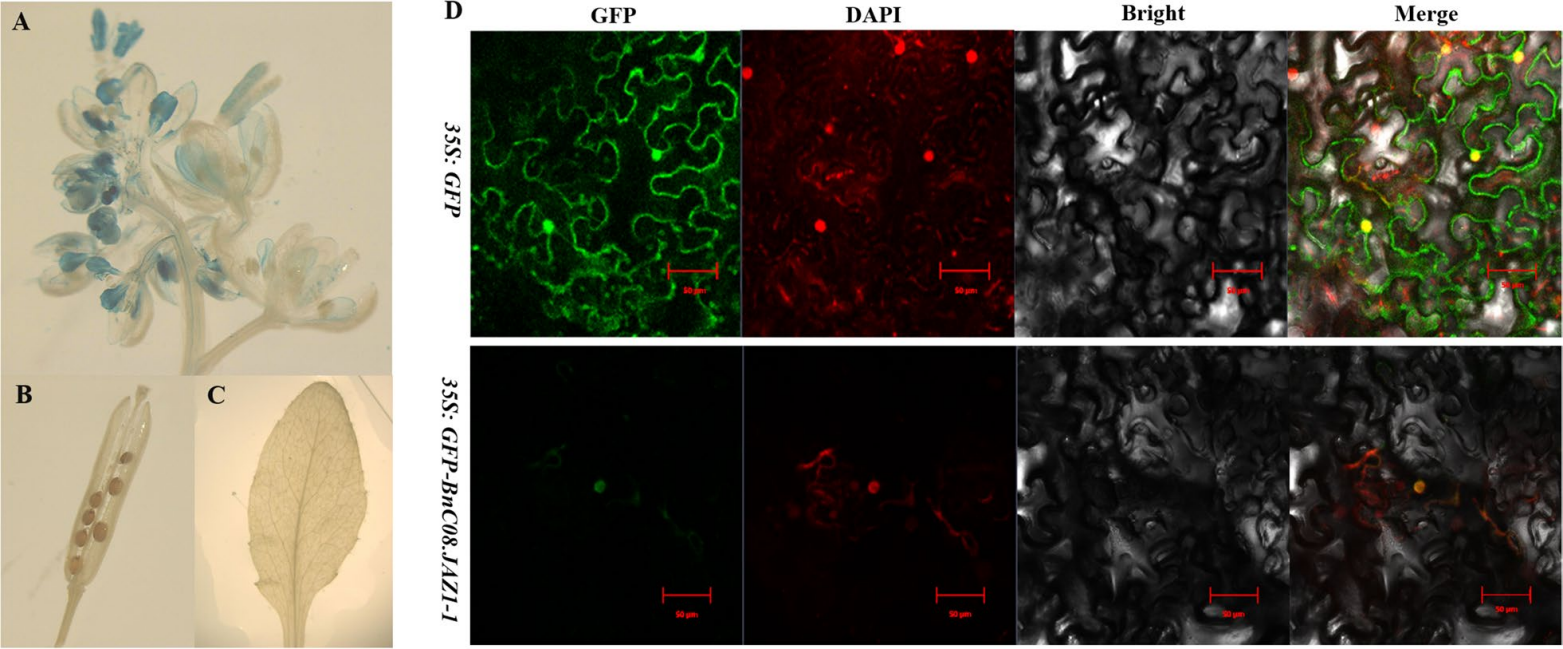
The tissue-specific expression and subcellular localization of BnC08.JAZ1-1 in *Arabidopsis*. **A** Histochemical analysis of GUS activity in *Arabidopsis* plants expressing *BnC08JAZ1pro::GUS*. **B** and leaf (**C**). **D** BnC08.JAZ1-1 protein was located in the nucleus of tobacco cells. GFP, green fluorescent protein; DAPI, 4′,6-diamidino-2-phenylindole

To further study the function of *BnC08.JAZ1-1*, its expression pattern in the different tissues of *Arabidopsis* was investigated by staining on selected p*BnC08.JAZ1-1*::*GUS* transgenic lines. The results showed that the GUS expression was mainly found in the vascular bundle and young flowers including petals, pistils, stamens, and developing ovules, but not in stem, leaf, mature silique, and seed (Fig. 7A–C). Therefore, the expression of *BnC08.JAZ1-1* was gradually decreased with the development and maturation of flowers. It was speculated that the expression level of *BnC08.JAZ1-1* gene might be related to the degree of organ development.

In addition, the subcellular localization of BnC08.JAZ1-1 was monitored by fusing to GFP protein. Vectors expressing *BnC08.JAZ1*-*1::GFP* or *GFP* alone were transferred into tobacco cells by the transient expression method, and the fluorescence was observed by confocal microscopy (Fig. 7D). The green fluorescence signal from *GFP* alone was distributed in both cell membrane and nucleus, while the green fluorescence signal from *BnC08.JAZ1-1::GFP* was completely coincident with the signal from DAPI nuclear dye, indicating that the BnC08.JAZ1-1 protein was localized in the nucleus, in line with the prediction (Table 1).

### Transcriptome analysis of *BnC08.JAZ1-1* overexpressing *Arabidopsis* seeds

To further investigate the downstream molecular mechanism underlying the increased seed weight in *BnC08.JAZ1-1* overexpression lines, the developing seeds of line OE-2 at 15 DAF were subjected to transcriptomic analysis, due to the fast seed filling at this stage [62, 63]. Statistics analysis of RAN-seq data showed that it met the standards and requirements for the following experiments (Additional file 6: Table S6). Using Hisat2 software, clean reads were efficiently and accurately mapped to genes. The FPKM method was adopted to calculate the relative expression of each gene, and a total of 16,541 expressed genes were detected. At the threshold of P < 0.05 and | log_2 fold change |≥ 1, a total of 582 differentially expressed genes (DEGs) were identified, including 123 (21%) up-regulated genes, and 459 (79%) down-regulated genes.

**Fig. 8:**
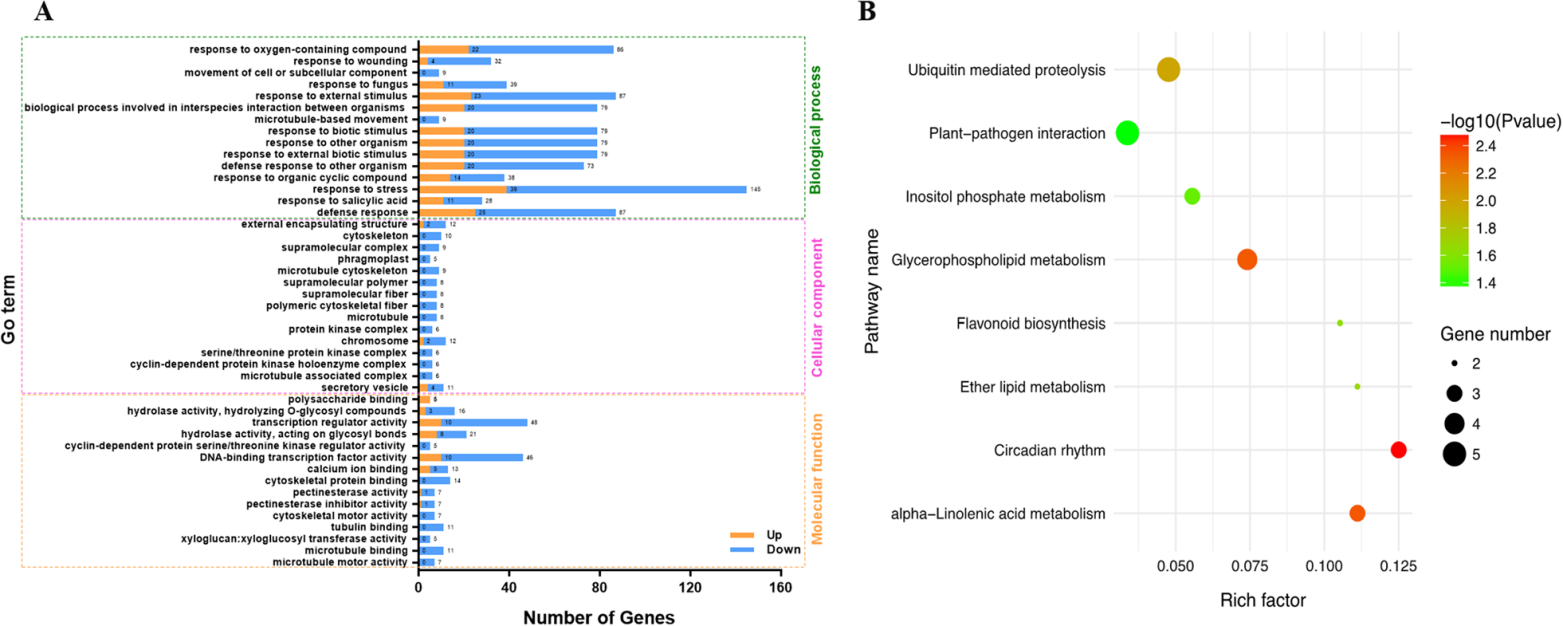
GO and KEGG enrichment analysis of differentially expressed genes (DEGs). **A** The horizontal and vertical axes show the – log10 (*P*-value) and enriched classes of DEGs. Orange columns: Upregulated DEGs. Blue columns: Downregulated DEGs. **B** The horizontal and vertical axes show the rich index and enriched pathway names, respectively. The top and bottom legends, respectively represent *P*-value and gene number

The GO enrichment analysis was performed for all 582 DEGs using Classification Super Viewer (Fig. 8A). From the perspective of biological processes, DEGs were most abundant in defense response, followed by transcription and signal transduction. This is understandable because the seed filling process involves cell proliferation and the expression of a series of genes, which also depends on the related signal transduction processes in the cell. Based on functionality, the most enriched category was hydrolytic enzyme activity, followed by transcription factor activity, and other enzyme activities. These activities were closely related to gene transcription regulation and seed filling. In terms of cellular components, the involvement of extracellular components was the most enriched, followed by the cell wall, nucleus, plasm membrane, chloroplast, and mitochondria.

Pathway enrichment analysis was conducted on all 582 DEGs (Fig. 8B and Additional file 7: Table S7A). They were significantly enriched in eight KEGG pathways: ubiquitin-mediated proteolysis, plant–pathogen interaction, circadian rhythm, stilbene compounds, biosynthesis of cellulosic (stilbenoid, diarylheptanoid, and gingerol biosynthesis), and glycerophospholipid metabolism (Additional file 7: Table S7B). During the development of seeds, the biosynthesis of stilbene compounds and inositol provides a material basis for the synthesis of many metabolites. Many previous studies have shown that the ubiquitin–proteasome pathway regulates seed size [62, 63, 64, 65, 66]. In the current study, five DEGs (*AT2G03190*↓, *AT3G21830*↓, *AT4G33270*↓, *AT2G25700*↓, *AT5G22920*↓) were enriched in ubiquitin-mediated proteolysis. It was speculated that the increased seed weight of *BnC08.JAZ1-1* overexpression lines might be through ubiquitin–proteasome pathways. In addition, four DEGs (*AT3G03530*↑, *AT3G03540*↑, *AT2G44810*↑, *AT4G01950*↓) were enriched in the phospholipid metabolism pathway, which was understandable as seed development is also a process of continuous accumulation and storage of lipids. Moreover, of all 582 DEGs, three were known genes in regulating seed weight, including *CYP78A9* (*AT3G61880*↑), *LEC2* (*AT1G28300*↓), and *ASPGB1* (*AT3G16150*↓) [67, 68].

## Discussion

As a member of the TIFY family, the JAZ subfamily plays a vital role in regulating plant growth, development, and the response of plants to abiotic and biotic stresses [31, 32, 33, 34]. JAZ proteins were found in all terrestrial plants, from the lowest mosses to higher dicotyledons. The JAZ subfamily proteins have been identified in many plant species including rice [50], wheat [51], maize [52], cotton [69], *B. rapa* [70], *B. oleracea* [71], *B. napus* [72], Chickpea [73], grape [74], soybean [53], rubber tree [75], *Salvia miltiorrhiza* [76], Moso bamboo [77] and sugarcane [78]. A total of 21, 22, and 52 JAZ proteins were identified in *B. rapa* cultivar Chiifu-401–42 [70], *B. oleracea* var. capitate lines (02–12 and D134) [71], and *B. napus* var. Damor [72]. In this study, 56, 28, and 31 *JAZ* genes were identified in *B. napus* (Zhongshuang11) as well as its two progenitors *B. rapa* (Chiffu V3.0) and *B. oleracea* (JZS V2.0), respectively. This showed a small inconsistency (about 10%) with the published result, which might be due to the different reference genomes and the different methods used for gene identification. First, all three reference genomes used in this study were the latest versions that were different from those used in the earlier published study. Second, in this study, the standard (have both TIFY and Jas motif) used to identify the *JAZ* genes was stricter than that used in the previous study. Thus, this represented an up-to-date and comprehensive structural analysis of JAZ members in *Brassica*.

In this study, the collinearity of *JAZ* genes was also analyzed in *Brassica* genus, using three representative species as examples. As expected, almost all of *JAZ*s in the An and Cn sub-genomes of *B. napus* had the syntenic genes in the diploid Ar and Co sub-genomes, respectively. This was understandable because of a very short evolutionary history (only 0.01 MYA) after the formation of *B. napus* from its progenitor [57]. Most of these gene pairs between the same sub-genomes (An and Ar or Cn and Co) had similar chromosomal locations. Interestingly, several gene pairs were located in the homologous chromosome segments from different sub-genomes, which should result from the homologous non-reciprocal translocations (HNRT) [79] or homologous exchanges [57, 80].

The previous studies showed that the *JAZ* subfamily genes in *B. rapa* were predominantly expressed in flower buds [70], while those in *B. oleracea* were highly expressed in roots [71]. In this study, the expression of 11 representative *BnJAZ* genes in Zhongshuang11 was basically consistent with the published database of gene expression in Zhongshuang11 (http://yanglab.hzau.edu.cn/) but different from those conducted using another *B. napus* cultivar Darmor [81]. Interestingly, the expression of these *BnJAZ* genes in Zhongshuang11 was similar to those of *BraJAZ*, no matter whether they were from the An or Cn sub-genomes. This was understandable as many genomic segments of Zhongshuang11 was introgressed from *B. rapa*, which was accordant with the breeding history of Chinese *B. napus* cultivars [82]. These results demonstrated that the patterns of *JAZ* gene expression varied in different accessions and species of *Brassica*. These 11 representative *BnJAZ* genes were highly induced by JA/MeJA in *B. napus*, a result similar to that reported in *B. rapa* [70], *B. oleracea* [71], and *B. napus* [72]. In the current study, most of the 11 representative *BnJAZ* were induced by cold and PEG treatment, which was basically consistent with a previous study in *B. rapa* [70].

The previous characterization of simple and high-order *jaz1* mutants revealed its diverse functions, and involved in growth (root, rosette, leaf senescence, hook curvature, flowering time) [12, 60, 61], reproductive development (fertility, seed germination, seed yield) [12, 60, 61, 83], and defense to biotic and abiotic stresses (pathogens, wound, cold, light, salt) [12, 60, 61, 84, 85, 86]. In the current study, the overexpression of *BnC08.JAZ1-1* showed earlier flowering, which was consistent with the delayed flowering time phenotype in *Arabidopsis jaz1* high-order mutant [12, 61]. The *BnC08.JAZ1-1* overexpression lines in *Arabidopsis* also displayed enlarged seeds, which might represent a new phenotype in *Arabidopsis* because it has not been reported in previous studies. Similarly, overexpression of *OsTIFY11b* which was in the same group as *JAZ1* also resulted in larger grains [83, 87]. These results suggested that *JAZ* family genes are also involved in the regulation of seed size/weight in plants. To elucidate the molecular mechanism of JAZ1, further experiment should be focused on its target genes (especially the DEGs regulating cell size), using the yeast one-hybrid or Chip-seq, etc.

## Conclusions

In the current study, we identified and characterized the JAZ subfamily in *B. napus*, including the analyses of phylogeny, cis-elements, protein motif, expression pattern, and response to abiotic stress and phytohormone treatment. The overexpression of *BnC08.JAZ1-1* in *Arabidopsis* suggested the positive role of JAZ1 in regulating flowering time and seed size/weight.

## Methods

### Plant materials and treatments

The oilseed rape variety ‘Zhongshuang11’ was planted in the Wuchang Experimental Base of Oil Crop Research Institute. Following a randomized complete block design, each plot contained three rows of 2 m long and 33 cm spacing, with 15 plants per row, three replicates. Root, stem, leaf, bud, petal, stamen, stigma, and silique were collected followed by immediately frozen in liquid nitrogen and stored at – 80 °C until analysis. Wild-type *Arabidopsis* (Columbia ecotype) and overexpression transgenic lines were planted in a growth room with constant temperature and light cycle (16 h light/8 h dark, 22 ± 1 °C, light intensity about 150 μmol/m^2^·s, humidity 60%).

To examine the pattern of *BnJAZ* expression in response to various stress treatments, the seedlings with the length of radicle about 5 mm (25 °C and 16 h/8 h light/dark cycle) were exposed to 0.15 M NaCl solution for salinity stress, 4 °C for cold stress on water-soaked filter paper, 15% PEG-6000 solution for drought stress, and waterflooding stress. The phytohormone treatments were performed as described in a previous study [88]. All these treatments were done with three biological replications.

### Identification of the JAZ proteins

Although there are 13 JAZ genes in *Arabidopsis*, the *AtJAZ13* was excluded in the current analysis due to its lack of the key TIFY domain [89]. Twelve AtJAZ protein sequences obtained from the TAIR website (http://www.arabidopsis.org/) were used as queries to identify their homologs in the public genomes of *B. rapa* (Chiifu_V3.0), *B. oleracea* (JZS_V2.0) and *B. napus* (Zhongshuang11_HZAU). Predicted JAZ proteins were then validated by HMMER using TIFY (PF06200) and Jas (PF09425) domains. Then, the JAZ proteins of HMM file were submitted to the Pfam database (http://pfam.xfam.org/) to recheck the two key domains. The threshold e-value for BLASTP was set to 1e-5 and the default restriction of HMMER was set to 0.01, respectively. In fact, the inclusion of AtJAZ13 in this analysis did not make a difference for the set of genes identified. Finally, 28 BrJAZs, 31BoJAZs, and 56 BnJAZs were obtained from the reference genomes of *B. rapa*, *B. oleracea,* and *B. napus*, respectively.

### Phylogenetic analysis of *JAZ* genes in *Brassicaceae*

To investigate the evolution of the *JAZ* gene subfamily in *Brassicaceae*, the protein sequences of the identified *JAZ* genes from *B. napus B. rapa*, *B. oleracea,* and *A. thaliana* were used to create a phylogenetic tree. The MEGA software was used to align these protein sequences. Then the neighbor-joining (NJ) method with 1000 bootstrap replicates was used to construct a phylogenetic tree.

To analyze the evolutionary constrictions of each *JAZ* gene pair, KaKs_Calculator 2.0 software was used to calculate the synonymous (Ks), non-synonymous (Ka) substitution, and Ka/Ks ratios [90].

### Gene structure, cis-acting element, and protein motif analysis

The PROTPARAM tool (http://web.expasy.org/protparam/) was used to estimate the physical and chemical characteristic of BnJAZ proteins, such as protein length, molecular weight, and isoelectric points. The WoLF PSORT server was adopted to predict the subcellular localization of BnJAZ proteins [91]. The MEME (V 4.11.4) was used to recognize the conserved motifs from 56 BnJAZ proteins. The PlantCARE webtool was used to predict the putative cis-elements within the 2 kb upstream of the start codons of 56 *BnJAZ* genes [92]. The cis-elements and protein motifs were displayed using TBtools (V 1.068).

### RNA extraction and gene expression analysis

The total RNA was extracted using the RNeasy Plant Mini Kit (QIAGEN). The reverse transcription was performed using the PrimeScript™ RT reagent Kit (Takara). qRT-PCR was performed as previously described [93]. The gene expression data were analyzed using the 2^−△△CT^ method as described previously [94]. The sequences of all primers used in this study are listed in Additional file 8: Table S8.

### Vector construction and plant transformation

To construct the overexpression vector, the CDS sequence of *BnC08.JAZ1-1* gene in the reference genome of Zhongshuang11 (http://cbi.hzau.edu.cn/cgi-bin/rape/download_ext) was used as a template to design primers (*BnC08.JAZ1-1* F/R). The cDNA of *BnC08.JAZ1-1* was amplified by KOD enzyme and then recombined into vector PD1301S using *Sac*I and *Kpn*I, constructing the recombinant plasmid p35S::*BnC08.JAZ1-1*. To construct the vector of *BnC08.JAZ1-1* promoter-driven *GUS* gene, the 2-kb upstream regulatory sequence of *BnC08.JAZ1-1* was amplified using the *BnC08.JAZ1-1* Pro_F/R to substitute the CaMV 35S promoter of pBI101 vector by T4 DNA ligase, generating *BnC08.JAZ1-1pro::GUS*. These plasmids were introduced into the *A. tumefaciens* strain GV3101 and transformed into *Arabidopsis* (Columbia ecotype).

To construct the GFP vectors, the pM999 plasmid was used as a template to obtain the GFP fragment (pM999GFP_F/R), and then it was ligated to the pD1301S vector. The construct GFP- PD1301S was used as a control of GFP alone. Next, using the constructed *BnC08.JAZ1-1*/pM999 vector as a template and the primers (*BnC08.JAZ1-1* GFP_F/R) were used to amplify the *BnC08.JAZ1-1*::*GFP* fusion fragment and the recovered product were digested with *Kpn*I and *Pst*I. The fusion fragment was then cloned into vector pD1301S.

### Phenotypic observation of *BnC08.JAZ1-1* overexpression *Arabidopsis*

The seed weight of *Arabidopsis* was measured using SC-G seed analyzer (Wanshen, China) which can identify the photographed seeds in a specific container and automatically count their number. The seed number per silique was examined from about ten well-developed siliques on the middle part of the main inflorescence. The seed weight was measured from the dried seeds that are threshed from the rest siliques. The seed number per silique and seed weight of each line were averaged from about ten plants.

### Subcellular localization and GUS histochemical localization of BnC08.JAZ1-1 protein

To detect the subcellular location of BnC08.JAZ1-1 protein, the generating *BnC08. JAZ1-1pro::GUS* plasmid was transferred into tobacco leaves followed the protocol described by Sparkes et al. [95]. To investigate the expression pattern of *BnC08.JAZ1-1*, *Arabidopsis* transgenic plants expressing *BnC08.JAZ1-1pro::GUS* were subjected to GUS staining. The plant tissues were completely immersed in the staining solution in a 37 °C incubator overnight. The samples were decolorized with 70% ethanol after staining. The expression pattern of *BnC08.JAZ1-1* was represented by the GUS stain location and intensity.

### Transcriptome analysis of *BnC08.JAZ1-1* overexpressing *Arabidopsis* seeds

The total RNA samples were isolated from the developing seeds at 15 DAF when the significant difference in seed weight occurred [63]. The quality of all RNA samples was checked using Nanodrop, with an A260/280 value between 1.81 and 2.16. The RNA samples were also subjected to gel electrophoresis to confirm the quality. The cDNA library was constructed and sequenced by Illumina HiSeqTM 2000 to produce raw sequence reads. Then raw reads were filtered, and clean reads were used for the assembly of sequence reads for subsequent analysis. The filtered clean reads were mapped on the *Arabidopsis thaliana* TAIR10 genome (http://www.arabidopsis.org/).

## Supplementary Information

**Additional file 1: Table S1.** The protein sequences of JAZ family genes in *A. thaliana*, *B. rapa*, *B. oleracea,* and *B. napus*.

**Additional file 2: Table S2.** A: Annotation of cis-elements in the promoter regions of *BnJAZ*s. B: Summary statistics of cis-elements in the promoter regions of *BnJAZ*s.

**Additional file 3: Table S3.** The information of identified 15 motifs in BnJAZ proteins.

**Additional file 4: Table S4.** The syntenic relationship of *JAZ* genes among *A. thaliana*, *B. rapa*, *B. oleracea,* and *B. napus*.

**Additional file 5: Table S5.** The information on gene duplication type, Ka, Ks, and Ka/Ks ratio values of *B. napus*.

**Additional file 6: Table S6.** The quality control of transcriptome sequence data.

**Additional file 7: Table S7.** A: 582 differentially expressed genes in *Arabidopsis*. B: The information on significant DEGs in the KEGG pathway.

**Additional file 8: Table S8.** A list of primers used for *BnJAZ* gene expression analysis.
